# Small but mighty: *OsKANADI1* and *OsYABBY5* regulate plant stature by tuning GA metabolism in rice

**DOI:** 10.1093/plcell/koae274

**Published:** 2024-10-17

**Authors:** Christian Damian Lorenzo

**Affiliations:** Assistant Features Editor, The Plant Cell, American Society of Plant Biologists; Center for Plant Systems Biology, VIB, Gent B-9052, Belgium; Department of Plant Biotechnology and Bioinformatics, Ghent University, B-9052 Gent, Belgium

Since the discovery of high-yielding, short-stature wheat and rice varieties during the Green Revolution, engineering plant height has been a major goal in several crops. However, plant height is a complex trait defined by several interacting players. Gibberellins (GAs) are known as the “green revolution hormones” due to their involvement in short-stature varieties, and they play major roles in promoting cell division, cell expansion, and ultimately defining plant height (Shani et al. 2024). The *YABBY* gene family is an important group of genes regulating many plant developmental processes, including organ growth and cell identity, and they also have been identified as regulators of plant height in dicotyledonous plants (Zhang et al. 2022). However, how *YABBY* genes regulate plant height in cereals, such as rice, and how this links to GA metabolism is a topic under research.

In new work, **Qi He and collaborators (He et al. 2024)** identified *OsKANADI1* (*OsKAN1*) as part of a regulatory hub controlling plant height in rice, together with *YABBY* and GA catabolism genes. The authors first mapped a mutation that causes dwarfism in a natural rice line (*oskan1*) to the *OsKAN1* locus. *OsKAN1* is a MYB transcription factor previously described to play roles in rice leaf development (Zhang et al. 2009), but no association had been made between this gene and plant height. By studying different natural alleles as well as generating *OsKAN1* CRISPR lines, the researchers confirmed that mutant *OsKAN1* lines produce shorter plants due to reduced internode length. External application of bioactive GA rescued the semi-dwarf phenotypes of these *oskan1* mutants, linking *OsKAN1* to GA signaling. Moreover, *OsKAN1* is highly expressed in internode tissue, and constitutive overexpression in transgenic lines (*OsKAN1ox*) resulted in increased bioactive GA levels and taller rice plants.

To elucidate the mechanism underlying the crosstalk between OsKAN1 and GAs, the authors performed a yeast 2-hybrid assay and identified OsYABBY5 (OsYAB5) as a possible interactor of OsKAN1. After validating the interaction though pull-down and BIFC assays (and considering they are both transcription factors), the authors explored the transcriptional regulation of the corresponding genes. RNA-seq of *OsKAN1*ox plants revealed reduced levels of *OsYAB5*. Subsequent reporter assays using the *OsYAB5* promoter driving *LUCIFERASE* (*LUC*) gene expression (*proOsYAB5::LUC*) confirmed that reporter activity decreased when *OsKAN1* was coexpressed. Intriguingly, the repression exerted by OsKAN1 over the promoter was abolished if both OsKAN1 and OsYAB5 were simultaneously coexpressed.

Going further to explore the mechanism behind *OsYAB5* regulation of plant height, constitutive expression of *OsYAB5* (*OsYAB5ox*) resulted in decreased bioactive levels of GA and semi-dwarf plants, while CRISPR loss-of-function mutants of *OsYAB5* displayed the opposite phenotype. Similar to *oskan1*, the application of bioactive GA rescued the dwarf phenotypes of *OsYAB5ox*. Transcriptomic analysis of the *OsYAB5ox* lines revealed that GA biosynthetic genes were mostly unaffected. However, GA catabolism genes, including members of the *OsGA2ox* family, were highly upregulated. Among them, *OsGA2ox6*, whose overexpression has previously shown to result in short-stature rice plants (Lo et al. 2008), was highly upregulated. Transient reporter assays using the *OsGA2ox6* promoter driving *LUC* expression established that OsYAB5 binds the promoter and induces *OsGA2ox6* expression and that coexpression of OsKAN1 alongside OsYAB5 nullified this induction, similar to the repressor activity of OsKAN1 on the *OsYAB5* promoter.

To shed more light on this complex regulation, the researchers developed *OsYAB5* ectopic expression lines driven by the *OsKAN1* promoter. These *proOsKAN1::OsYAB5* plants displayed increased height compared with controls, in contrast with *OsYAB5* constitutive expression lines. Furthermore, this same *proOsKAN1::OsYAB5* transgene in the *oskan1* background resulted in severely shortened plants. As *OsYAB5* and *OsGA2ox6* expression is high in node tissue in wild-type plants, the authors hypothesized that the formation of the protein complex negatively regulates GA signaling and elongation in nodes, while at internodes (the expression domain of *OsKAN1*), OsYAB5 protein is repressed and *OsKAN1* inhibition results in enlarged internodes, ultimately through *OsGA2ox6* downregulation (see Fig.).

**Figure.**
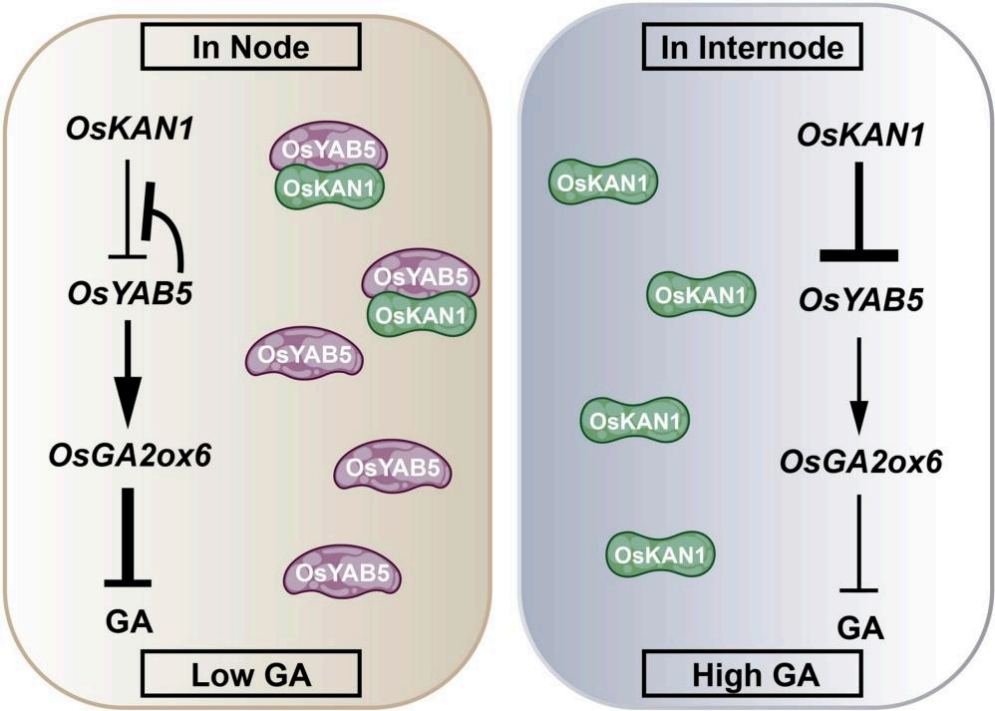
Schematic depiction of the interactions and regulatory relationships of *OsKAN1-OsYAB5-OsGA2ox6* in node and internode. Reprinted from He et al. (2024), Figure 8.

Overall, the results obtained by the group elucidated a novel regulatory hub involving OsKAN1, OsYAB5, and OsGA2ox6, acting both at transcription and protein levels to regulate plant height in rice.

## Data Availability

None to declare.
